# Magnetic Hyperthermia Method Synthesis of Water-Soluble Silicon–Carbon Dots: Excitation-Independent Fluorescence Materials

**DOI:** 10.3390/molecules30061222

**Published:** 2025-03-09

**Authors:** Bing-Yu Li, Chun-Yan She, Jun-Chao Deng, Wen-Ming Shu, Wei-Chu Yu

**Affiliations:** Hubei Engineering Research Centers for Clean Production and Pollution Control of Oil and Gas Fields, College of Chemistry and Environmental Engineering, Yangtze University, Jingzhou 434023, China; lbyytzeu@126.com (B.-Y.L.); scyyangtzeu@126.com (C.-Y.S.); djcyangtzeu@126.com (J.-C.D.)

**Keywords:** silicon–carbon dots, silica microspheres, excitation independent, magnetic hyperthermia method, fluorescent markers

## Abstract

Carbon dots (CDs) have attracted widespread attention in recent years due to their synthetic simplicity, biocompatibility, and unique photoluminescent behavior. In this work, water-soluble silicon–carbon dots (SiCDs) were synthesized, and their properties were evaluated. First, a series of SiCDs was prepared by using a novel magnetic hyperthermia method from citric acid (CA) and 3-(2-aminoethylamino) propyldimethoxymethylsilane (AEAMPS). Then, based on the Stöber method, silica (SiO_2_) was loaded onto the SiCDs in a one-pot reaction to obtain SiCDs@SiO_2_ microspheres. This synthesis strategy is safe, efficient, and simple, allowing gram-scale production in a short time. The resulting SiCDs@SiO_2_ microspheres exhibited excellent fluorescent performance, along with high water solubility and independence of excitation fluorescence. The SiCDs@SiO_2_ microspheres possessed good thermal resistance and acid–base stability. The influence of storage time and different metal ions on the microsphere suspension was minimal. The SiCDs@SiO_2_ microspheres show potential applications for water detection in horizontal wells as fluorescent markers.

## 1. Introduction

Carbon dots (CDs), also known as carbon quantum dots, are zero-dimensional carbon-based nanomaterials composed of dispersed quasi-spherical carbon particles with a diameter smaller than 10 nm [1]. CDs possess attractive features such as good water solubility, high biocompatibility, low toxicity, abundant sources, and favorable fluorescent properties. Consequently, CDs have been widely applied in various fields, including biomedicine [2], catalysis, and chemical sensing [3,4].

Various synthesis methods have been developed based on the characteristics of CDs for different applications [5,6]. According to the carbon source, the synthesis strategies for CDs can be mainly categorized into two approaches: “top-down” and “bottom-up” [7,8]. The top-down approach involves using methods such as laser ablation, arc discharge, and electrochemical oxidation to decompose larger carbon structures into smaller ones. However, these methods have unavoidable drawbacks, including complex removal of strong acids and oxidants, potential environmental pollution, and providing CDs with limited surface functional groups [9]. These problems can be addressed through the bottom-up approach, which involves the dehydration carbonization of small-molecule carbon precursors using techniques such as microwave heating, microfluidics, hydrothermal/solvothermal methods, and magnetic hyperthermia [10]. Although traditional pyrolysis and hydrothermal/solvothermal methods can synthesize various functional CDs, they are energy intensive and time consuming, making it challenging to meet the demands for large-scale production. In contrast, the magnetic hyperthermia method [11,12] offers advantages such as simple operation and short reaction times. Magnetic hyperthermia has been widely used in the synthesis of materials as a heat source capable of enhancing energy generation and diffusion under electromagnetic fields [13]. Under an appropriate alternating magnetic field (AMF), magnetic nanoparticles (MNPs) are capable of generating heat, which externally triggers a burst of chemical reactions. This method not only provides CDs with high water solubility and fluorescence quantum yield (QY) but also enables large-scale preparation in a short period [14]. Thus, the magnetic hyperthermia method is a valuable approach for CD synthesis and can promote the industrialization of CDs.

Most CDs exhibit excitation wavelength-dependent behavior, where the emission wavelength varies with the excitation wavelength. This excitation-wavelength dependence may originate from the influence of surface states of the CDs [15]. Addressing the excitation-wavelength dependence of CDs is garnering increasing attention from researchers. One approach to mitigate this problem involves encapsulating CDs in silica (SiO_2_) to form silicon–carbon dots (SiCDs), which alters the energy levels of traps on the surface of the CDs. The surface passivation induced by the Si-O-C bonds increases the stability of fluorescence emission from embedded CDs, rendering it independent of the excitation wavelength. Such independence from excitation wavelength leads to improved absorption and high QY [16]. The stable fluorescent properties of SiCDs facilitate reliable detection and make them well-suited for use as markers.

Herein, we prepare a series of SiCDs via the magnetic hyperthermia method, in which citric acid (CA) serves as the carbon source and 3-(2-aminoethylamino) propyldimethoxymethylsilane (AEAMPS) as the passivation nitrogen source. Different SiCDs are produced by varying precursors. A one-pot approach based on the Stöber method [17] is then employed to load the SiCDs on SiO_2_ microspheres, resulting in the formation of SiCDs@SiO_2_ spheres. This synthetic strategy is safe, efficient, and straightforward, enabling gram-scale production in a short time. The fluorescent properties and water solubility of the SiCDs@SiO_2_ microspheres are evaluated. The stability of SiCDs@SiO_2_ is investigated by conducting a series of property tests assessing thermal stability, stability in fracturing fluids, the influence of pH, and potential interference from metal ions. The synthesized SiCDs@SiO_2_ microspheres exhibit outstanding properties and can potentially be used as markers for water detection in horizontal wells.

## 2. Results and Discussion

### 2.1. Preparation of Water-Soluble SiCDs

**Figure 1:**
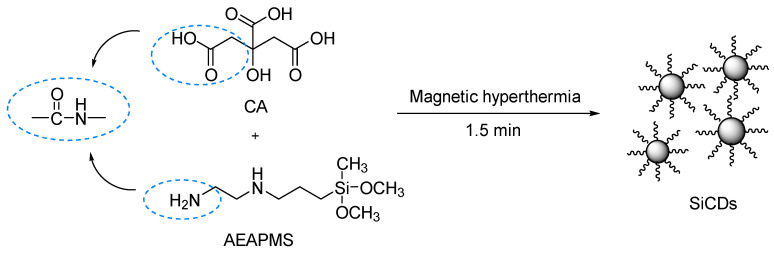
Schematic diagram of the synthesis of SiCDs-1.

**Figure 2:**
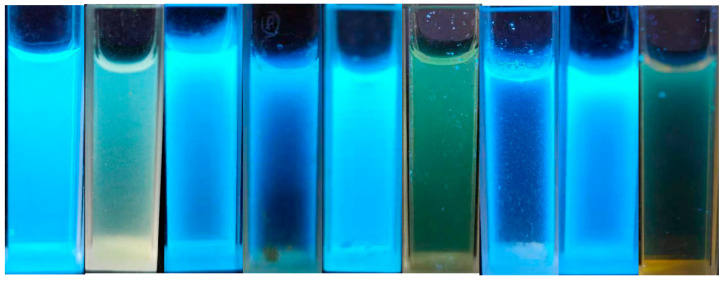
Luminescence of aqueous solutions of SiCDs under 365 nm UV irradiation (SiCDs-1–SiCDs-9).

To prepare CD-embedded SiO_2_ microspheres, water-soluble SiCDs were first synthesized rapidly by magnetic hyperthermia using CA as the carbon source. AEAPMS provided -NH_2_ groups for the amidation reaction that passivated the CD surfaces and allowed silane grafting (Figure 1). The grafted -Si-O-CH_3_ groups could be hydrolyzed to Si-OH, which ensured that the surface of the resulting SiCDs was enriched in Si-OH. In this case, the enriched Si-OH groups not only endow the SiCDs with good water solubility but also allow them to be embedded into the Si-O network during the formation of SiO_2_ microspheres via the Stöber reaction. By changing the precursors, different luminescent SiCDs were prepared (Figure 2). The resulting silane-functionalized CDs were emissive both in solution and in the solid state. Among the nine SiCD samples, SiCDs-1 and SiCDs-2 attracted our interest because of their unique photoluminescence properties, high yields, and favorable fluorescence behavior. SiCDs-1 displayed the highest QY (about 63%) of the samples and emitted bright blue light, whereas the QY of SiCDs-2 was about 22%. Therefore, SiCDs-1 and SiCDs-2 were selected for further study.

#### 2.1.1. Optical Performance Characterization of Water-Soluble SiCDs

**Figure 3:**
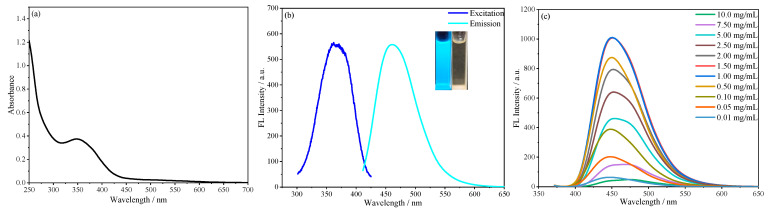
(**a**) UV–vis absorption and (**b**) fluorescence spectra of SiCDs-1. The insets show photographic images of SiCDs-1 under UV irradiation (**left**) and natural light (**right**). (**c**) Fluorescence spectra of aqueous solutions of SiCDs-1 with different concentrations.

**Figure 4:**
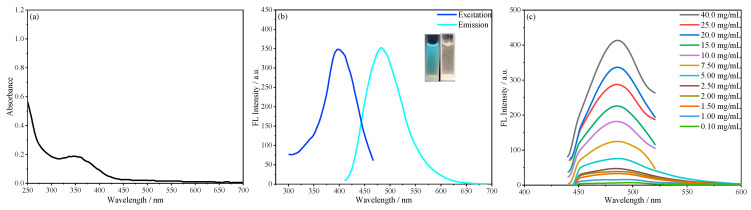
(**a**) UV–vis absorption and (**b**) fluorescence spectra of SiCDs-2. The insets show photographic images of SiCDs-2 under UV (**left**) and natural light (**right**). (**c**) Fluorescence spectra of aqueous solutions of SiCDs-2 with different concentrations.

The fluorescence properties of SiCDs-1 and SiCDs-2 were systematically characterized. Both SiCDs exhibited a UV–vis absorption peak at 350 nm (Figure 3a and Figure 4a), likely attributed to the n−π* transition of C=O bonds. Under natural light, their aqueous solutions appeared yellowish and transparent (insets on the right of Figure 3b and Figure 4b). However, distinct fluorescence colors were observed under 365 nm UV irradiation: SiCDs-1 emitted bright blue light (Figure 3b, left inset), while SiCDs-2 showed a blue-green emission (Figure 4b, left inset).

The excitation and emission profiles further differentiated the two samples. For SiCDs-1, the fluorescence excitation peak was centered at 365 nm, with an emission maximum of 460 nm (Figure 3b). In contrast, SiCDs-2 displayed a redshifted excitation peak at 400 nm and a corresponding emission peak at 483 nm (Figure 4b).

Concentration-dependent fluorescence studies revealed similar trends but distinct behaviors. When excited at 360 nm (SiCDs-1) or 400 nm (SiCDs-2), the emission intensity of both samples initially increased with concentration, reaching a maximum before declining at higher concentrations for SiCDs-1 (Figure 3c). Notably, SiCDs-2 maintained a fixed emission center wavelength (483 nm) across all concentrations despite intensity variations (Figure 4c).

#### 2.1.2. Structural Characterization of Water-Soluble SiCDs

**Figure 5:**
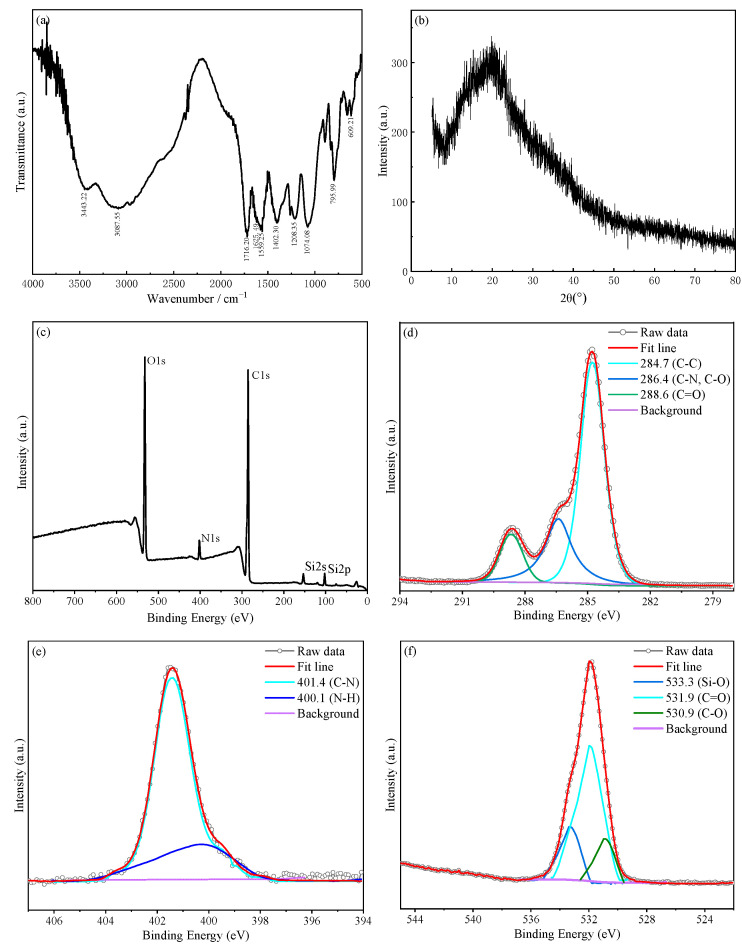
(**a**) FTIR spectrum of SiCDs-1. (**b**) XRD pattern of SiCDs-1. (**c**) XPS survey spectrum of SiCDs-1. (**d**) C 1s, (**e**) N 1s, and (**f**) O 1s spectra of SiCDs-1.

The chemical composition and structure of the SiCDs were analyzed using FTIR spectroscopy, X-ray diffraction (XRD), and X-ray photoelectron spectroscopy (XPS). Figure 5a depicts the FTIR spectrum of SiCDs-1, which contains absorption peaks corresponding to characteristic molecular vibrations [18]. The broad peak at 3443 cm^−1^ indicates the presence of N-H and O-H groups, which provide the basis for the high water solubility of the SiCDs. The absorption band at 1625 cm^−1^ is a characteristic peak of the amide bond (O=C−NH) due to the C=O stretching vibration. The presence of amide bonds was further confirmed by the observation of N-H stretching and bending vibrations at 3443 and 1559 cm^−1^, and the C-N stretching vibration at 1208 cm^−1^. Another important finding is the presence of Si-C and Si-O-C characteristic peaks at 795 and 1074 cm^−1^, respectively. These peaks prove the passivation effect of the amidation reaction on the CD surface and indicate that AEAPMS was grafted onto the CD surface. The results of FTIR spectral analysis were supported by those obtained by XRD [19]. The XRD pattern in Figure 5b shows a broad peak centered at 2θ = 21°, which indicates that the turbostratic carbon structure possesses a nanocrystalline structure. The XPS results of SiCDs-1 are shown in Figure 5c–f [20]. The full spectrum analysis shows that SiCDs-1 contains the elements C, N, O, and Si, which further confirms the grafting of AEAPMS on the CD surface (Figure 5c). Split-peak fitting of the C 1s spectrum provided three characteristic peaks located at 284.7, 286.4, and 288.6 eV, which corresponded to C-C, C-N/C-O, and C=O groups, respectively (Figure 5d). N 1s spectra (Figure 5e) revealed the presence of C-N and N-H groups. Split-peak fitting of the O 1s spectrum (Figure 5f) demonstrated the presence of three distinct peaks at 530.9, 531.9, and 533.3 eV, which indicated the presence of C-O, C=O, and Si-O groups, respectively. The XPS results are consistent with those of FTIR spectroscopy, revealing a rich distribution of functional groups on the surface of SiCDs-1 and providing evidence for the passivating amidation reaction on the SiCDs-1 surface and the presence of Si-O-CH_3_.

**Figure 6:**
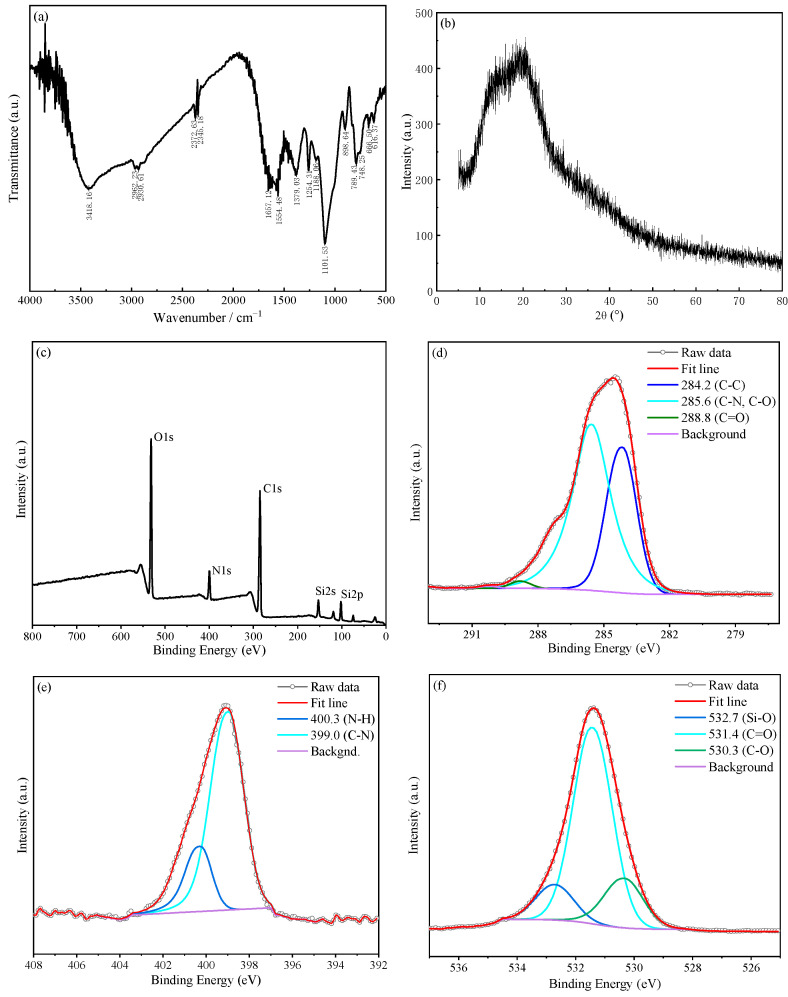
(**a**) FTIR spectrum of SiCDs-2. (**b**) XRD pattern of SiCDs-2. (**c**) XPS survey spectrum of SiCDs-2. (**d**) C 1s, (**e**) N 1s, and (**f**) O 1s spectra of SiCDs-2.

Figure 6a shows the FTIR spectrum of SiCDs-2, which has a broad peak at 3418 cm^−1^ that indicates the presence of N-H and O-H groups, providing the basis for the excellent water solubility of SiCDs-2. The absorption band at 1657 cm^−1^ is the characteristic peak of the amide bond (O=C−NH) due to the C=O stretching vibration. The presence of amide bonds was further confirmed by the presence of N-H stretching and bending vibrations at 3418 and 1554 cm^−1^, and the C-N stretching vibration at 1254 cm^−1^. Si-C and Si-O-C characteristic peaks were observed at 789 and 1101 cm^−1^, respectively, confirming the passivation effect of the amidation reaction on the CD surface and indicating that AEAPMS was successfully grafted on the CD surface. The XRD pattern of SiCDs-2 in Figure 6b shows a broad peak centered at 2θ = 21°, which is assigned to the presence of nanocrystals in the turbostratic carbon structure. Figure 6c–f shows the XPS results of SiCDs-2. The full spectrum analysis (Figure 6c) indicates that SiCDs-2 contains C, N, O, and Si, which further proves that AEAPMS was grafted on the CD surface. The C 1s spectrum was fitted with peaks located at 284.2, 285.6, and 288.8 eV, which correspond to C-C, C-N/C-O, and C=O groups, respectively (Figure 6d). The N 1s spectrum (Figure 6e) revealed the presence of C-N and N-H groups. Split-peak fitting of the O 1s spectrum revealed three peaks located at 530.3, 531.4, and 532.7 eV, indicating the presence of C-O, C=O, and Si-O groups, respectively (Figure 6f). The XPS results agree with those of FTIR spectroscopic analysis, revealing the rich functional group distribution on the surface of SiCDs-2 and providing evidence for the passivating amidation reaction on the SiCDs-2 surface and the presence of Si-O-CH_3_.

### 2.2. Preparation of Water-Soluble SiCDs@SiO_2_ Spheres and Pure SiO_2_ Spheres

**Figure 7:**
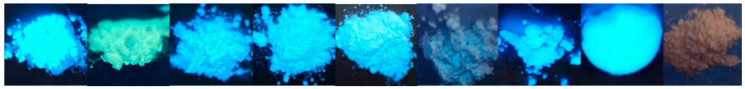
Luminescence of different SiCDs@SiO_2_ microspheres under 365 nm UV light (SiCDs@SiO_2_-1–SiCDs@SiO_2_-9 from left to right).

The SiCDs (SiCDs-1–SiCDs-9) were used to prepare fluorescent microspheres (SiCDs@SiO_2_) with different emission colors, as shown in Figure 7. Most of the SiCDs@SiO_2_ samples exhibited blue fluorescence under 365 nm irradiation, among which SiCDs@SiO_2_-1 showed intense bright blue fluorescence and SiCDs@SiO_2_-2 displayed bright green emission. Because of their unique photoluminescence properties, high yields, and good fluorescence efficiency, SiCDs@SiO_2_-1 and SiCDs@SiO_2_-2 were chosen for further study.

#### 2.2.1. Optical Performance Characterization of Water-Soluble SiCDs@SiO_2_ Spheres

**Figure 8:**
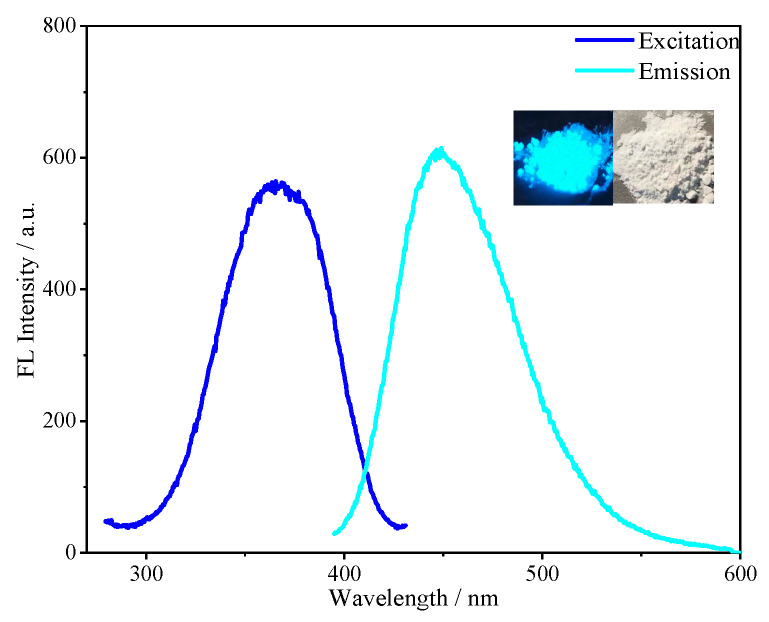
Fluorescence spectra of SiCDs@SiO_2_-1. The insets show photographic images of SiCDs@SiO_2_-1 under UV irradiation (**left**) and natural light (**right**).

The optical properties of the fluorescent microspheres were examined by measuring fluorescence emission spectra. The FL spectra of SiCDs@SiO_2_ spheres with the same luminescent properties as those of the corresponding SiCDs are shown in Figure 8. Under 365 nm UV irradiation, SiCDs@SiO_2_-1 exhibited strong blue emission (left inset of Figure 8). Under natural light, SiCDs@SiO_2_-1 was observed as a white powder (right inset of Figure 8). The excitation wavelength maximum of SiCDs@SiO_2_-1 appeared around 360 nm, and its emission peak was centered around 460 nm.

**Figure 9:**
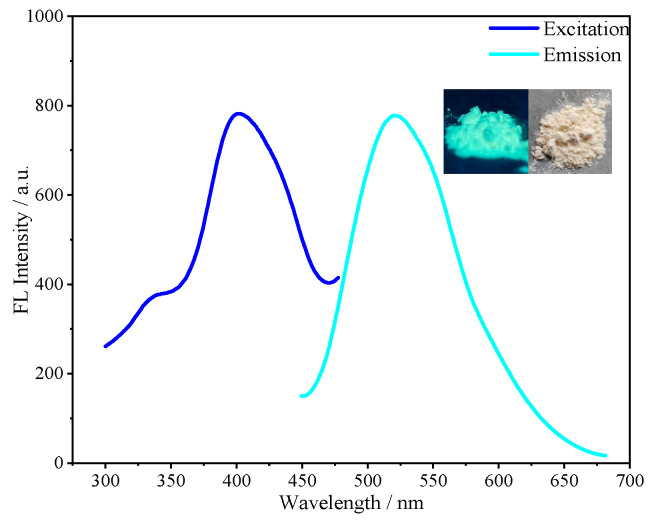
Fluorescence spectra of SiCDs@SiO_2_-2. The insets show photographic images of SiCDs@SiO_2_-2 under UV irradiation (**left**) and natural light (**right**).

As shown in Figure 9, under the 365 nm UV irradiation, SiCDs@SiO_2_-2 exhibited green emission. Under natural light, SiCDs@SiO_2_-2 was a yellowish powder. The excitation peak of SiCDs@SiO_2_-2 was observed around 400 nm, and its emission peak center appeared around 520 nm.

#### 2.2.2. Morphology and Structural Characterization of Water-Soluble SiCDs@SiO_2_ Spheres

**Figure 10:**
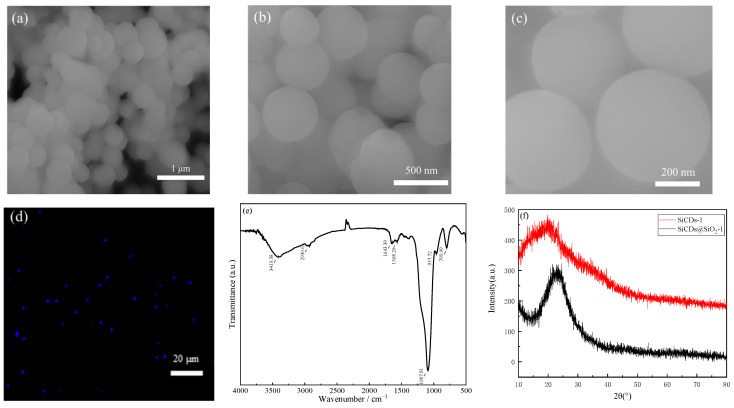
(**a**–**c**) TEM images of the SiCDs@SiO_2_-1 microspheres. (**d**) Laser confocal microscopy image of SiCDs@SiO_2_-1 spheres. (**e**) FTIR spectrum of SiCDs@SiO_2_-1 spheres. (**f**) XRD pattern of SiCDs@SiO_2_-1 spheres.

The surface morphology of the fluorescent microspheres was examined by FESEM and fluorescence microscopy. As shown in Figure 10a–c, SiCDs@SiO_2_-1 particles were spherical with smooth surfaces, a uniform size of about 500 nm, and high stability. Figure 10d depicts a laser confocal microscope image of SiCDs@SiO_2_-1, which emitted blue fluorescence at λ = 405 nm, indicating that the SiCDs are embedded in the SiO_2_ spheres, and each microsphere is an individual luminescent body. The internal structure of the SiCDs@SiO_2_ microspheres is complex because of the presence of Si-O-CH_3_ and abundant Si-OH groups on the SiCDs surface. The SiCDs are connected to form a Si-O network in the SiO_2_ spheres by Si-OH dehydration and condensation catalyzed by ammonium hydroxide. In this case, a SiO_2_/SiCD middle layer was grown on pure SiO_2_ spheres. The chemical composition and structure of SiCDs@SiO_2_-1 were analyzed using XRD and FTIR spectroscopy. Figure 10e shows the FTIR spectrum of SiCDs@SiO_2_-1. The extremely strong peak at 1087 cm^−1^ was characteristic of Si-O-C; the peaks at other positions were weaker, which indicates that SiCDs-1 was connected to the Si-O network of the SiO_2_ spheres. That is, the FTIR spectrum of SiCDs@SiO_2_-1 demonstrates that the SiCDs were encapsulated in the SiO_2_ spheres. Although the SiO_2_ spheres prepared by Stöber’s method had typical amorphous features and their XRD pattern was characterized by broad peaks (Figure 10f), embedding SiCDs-1 in SiO_2_ did not affect the physical phase of the SiO_2_ spheres. The content of CDs was very small compared with that of SiO_2_, so peaks from CDs were not detected in the XRD pattern of SiCDs@SiO_2_-1.

**Figure 11:**
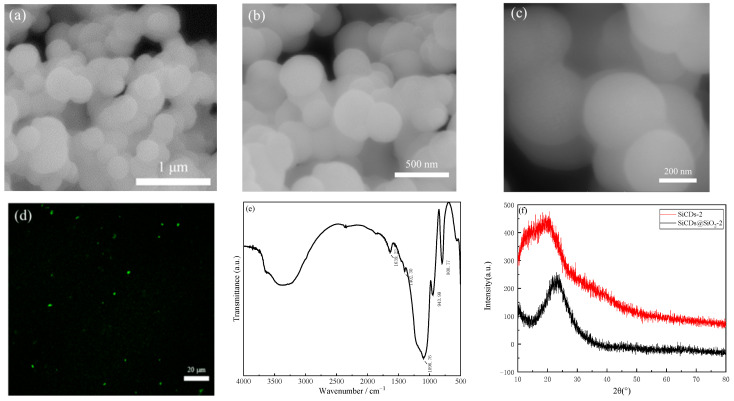
(**a**–**c**) TEM images of the SiCDs@SiO_2_-2 microspheres. (**d**) Laser confocal microscopy image of SiCDs@SiO_2_-2 spheres. (**e**) FTIR spectrum of SiCDs@SiO_2_-2 spheres. (**f**) XRD pattern of SiCDs@SiO_2_-2 spheres.

As shown in Figure 11a–c, SiCDs@SiO_2_-2 particles were spherical with smooth surfaces and a uniform size of about 400 nm. Figure 11d presents a laser confocal microscopy image of SiCDs@SiO_2_-2, which emitted green fluorescence at λ = 514 nm, indicating that the SiCDs were embedded in the SiO_2_ spheres, and each microsphere was an independent luminescent body. The chemical composition and structure of SiCDs@SiO_2_ were further analyzed by XRD and FTIR spectroscopy. Figure 11e shows the FTIR spectrum of SiCDs@SiO_2_-2, which is similar to that of SiCDs@SiO_2_-1, with an intense peak at 1096 cm^−1^, which is a characteristic peak of Si-O-C. The peaks at other positions are weaker, which indicates that SiCDs-2 was integrated into the Si-O network of the SiO_2_ spheres. Figure 11f shows the XRD pattern of SiCDs@SiO_2_-2, which indicates that embedding SiCDs-2 did not change the physical phase of the SiO_2_ spheres. The content of CDs was very small compared to that of SiO_2_, so peaks from the CDs were not detected in the XRD pattern of SiCDs@SiO_2_-2.

#### 2.2.3. Excitation Wavelength Independence of CDs Fluorescence

Most CDs emit blue fluorescence under UV light and exhibit excitation wavelength-dependent behavior; that is, as the excitation wavelength changes, the wavelength and intensity of the emitted light also change. Currently, there are several explanations for the excitation wavelength dependence of the fluorescence of CDs. For example, a wide particle size distribution of CDs will lead to multiple energy gaps, which will cause multiple transition modes and result in excitation wavelength-dependent fluorescence. On the contrary, when the particle size distribution of CDs is narrow, the band gap dominated by quantum-limited effects tends to a single value, thus reducing the excitation wavelength dependence [21,22]. This result further confirms that the optical properties of CDs can be effectively regulated by controlling the uniformity of particle size distribution. The distribution of different surface states is related to the functional groups on the CDs surface because different functional groups lead to the existence of multiple emission centers. In this scenario, at a certain excitation wavelength, certain emission centers will dominate [23]. The formation of carbon nuclei may also affect the dependence of CDs fluorescence on excitation wavelength [24]. It is generally believed that the excitation wavelength-dependent behavior of CDs originates from the action of one or more of the above factors.

**Figure 12:**
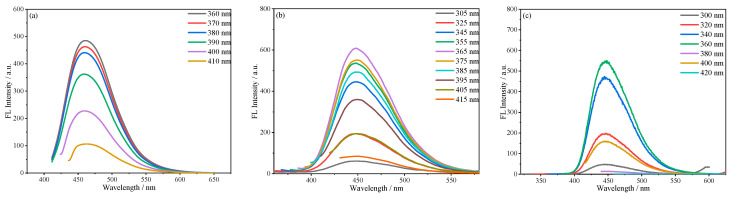
Excitation wavelength-dependent photoluminescence of (**a**) SiCDs-1, (**b**) SiCDs@SiO_2_-1, and (**c**) RSiCDs-1.

**Figure 13:**
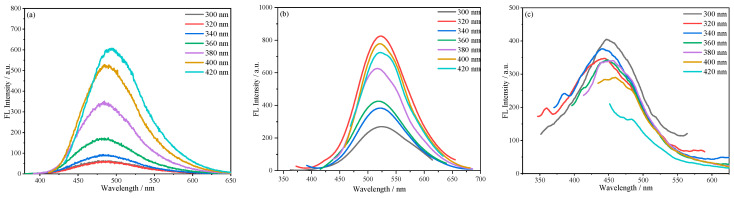
Excitation wavelength-dependent photoluminescence behavior of (**a**) SiCDs-2, (**b**) SiCDs@SiO_2_-2, and (**c**) RSiCDs-2.

The fluorescence spectra of SiCDs, SiCDs@SiO_2_, and RSiCDs at different excitation wavelengths were measured (Figure 12 and Figure 13). Figure 12a presents the fluorescence emission spectra of SiCDs-1 solutions of the same concentration at different excitation wavelengths. Unlike most CDs, when the excitation wavelength was varied in the range of 360–410 nm, the fluorescence intensity decreased substantially with the increase in excitation wavelength, but the fluorescence emission peak of SiCDs-1 around 460 nm did not show obvious displacement, reflecting the excitation wavelength-independent emission characteristics of SiCDs-1. The excitation wavelength-independent fluorescence properties of CDs have been reported by some researchers [25]. However, these CDs usually require a complicated purification process to display fluorescence independent of excitation wavelength. Usually, the excitation-dependent fluorescence properties of CDs are caused by the distribution of different particle sizes and surface states [26]. The excitation wavelength-independent fluorescence properties of SiCDs synthesized by a magnetic hyperthermia method in this paper without any further purification may indicate that the particle size and surface states of SiCDs have fairly uniform distributions. Furthermore, the energy transfer in the magnetic hyperthermia reaction process increases the yield compared to that of CDs prepared by the conventional method. The resulting CDs display bright emission under UV light. Figure 12b shows the fluorescence emission spectra of SiCDs@SiO_2_-1 at different excitation wavelengths. When the excitation wavelength is in the range of 305–365 nm, the fluorescence intensity increases markedly with the excitation wavelength. The fluorescence intensity is the strongest at an excitation wavelength of 365 nm. In the range of 365–415 nm, the fluorescence intensity of SiCDs@SiO_2_-1 decreases substantially with rising excitation wavelength. However, the fluorescence emission peak of SiCDs@SiO_2_-1 at about 448 nm does not shift with the change in excitation wavelength, which reflects its excitation wavelength-independent emission characteristics. When the SiO_2_ layer was etched away to release the SiCDs, i.e., RSiCDs-1, the emission peak around 446 nm remained unchanged when the excitation wavelength was increased from 300 to 420 nm (Figure 12c).

Similarly, as shown in Figure 13a, the fluorescence emission spectra of SiCDs-2 solutions of the same concentration were measured at different excitation wavelengths. When the excitation wavelength was in the range of 320–420 nm, the fluorescence intensity increased markedly with the excitation wavelength, but the emission peak did not shift, reflecting the excitation wavelength-independent emission characteristics of SiCDs@SiO_2_-2. Figure 13b presents the fluorescence emission spectra of SiCDs@SiO_2_-2 at different excitation wavelengths. When the excitation wavelength was in the range of 300–420 nm, the fluorescence emission peak located at about 520 nm did not display obvious displacement, consistent with excitation wavelength-independent emission characteristics. For RSiCDs-2 obtained by etching away the SiO_2_ layer, when the excitation wavelength was increased from 300 to 420 nm, only a small shift of the emission center was observed, which was different from the typical excitation wavelength-dependent fluorescent behavior of CDs (Figure 13c).

Because SiCDs@SiO_2_ showed almost the same fluorescence spectrum as those of SiCDs and RSiCDs, and pure SiO_2_ microspheres do not fluoresce, the fluorescence of SiCDs@SiO_2_ must be related to the SiCDs inside the SiO_2_ microspheres. Mechanistically, the photoluminescence of CDs can be attributed to the presence of stable surface energy-level traps after surface passivation, which are mainly related to carboxyl, amino, and hydroxyl groups. The surface states of CDs strongly affect their spectral properties. The fluorescence of our SiCDs does not depend on excitation wavelength, which is probably caused by the relatively uniform surface of the CDs and small variety of functional groups. The requirement for surface passivation to become photoluminescent is shared by both CDs and SiO_2_ nanocrystals, and a large specific surface area is necessary for the surface-passivated particles to exhibit strong photoluminescence [27]. In addition, studies have shown that the fluorescence of carbon–SiO_2_ composites is related to Si-O-C bonding and that the formation of Si-O-C on the surface of SiCDs strongly contributes to the increase in fluorescence intensity [28].

### 2.3. Properties of Water-Soluble SiCDs@SiO_2_ Spheres

#### 2.3.1. Thermal Stability of Microsphere Suspensions

**Figure 14:**
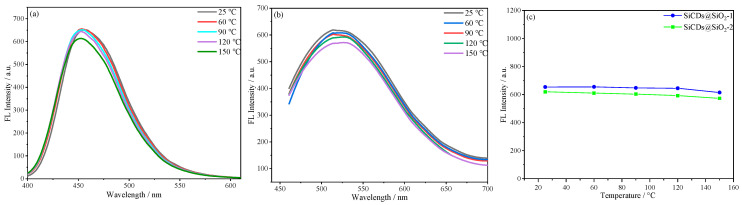
Fluorescence spectra of (**a**) SiCDs@SiO_2_-1 and (**b**) SiCDs@SiO_2_-2 microsphere suspensions (0.2 mg/mL) at different temperatures (25–150 °C). (**c**) Relationship between temperature and fluorescence emission intensity for SiCDs@SiO_2_-1 and SiCDs@SiO_2_-2.

As illustrated in Figure 14a,b, the fluorescence intensities of SiCDs@SiO_2_-1 and SiCDs@SiO_2_-2 demonstrated minimal variation at temperatures between 25 and 150 °C, indicating that the microsphere suspensions remained stable. The graph of fluorescence intensity versus temperature in Figure 14c reveals that the fluorescence intensity of the microspheres decreased only slightly with rising temperature. Overall, the prepared fluorescent microspheres possess high thermal stability.

#### 2.3.2. Effect of pH on the Emission Behavior of Microsphere Suspensions

**Figure 15:**
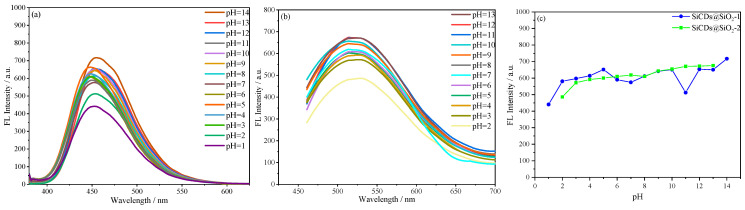
(**a**) Fluorescence intensity of SiCDs@SiO_2_-1 suspensions (0.2 mg/mL) at different pH (1–14). (**b**) Fluorescence intensity of SiCDs@SiO_2_-2 suspensions (0.2 mg/mL) at different pH (2–13). (**c**) Relationship between fluorescence emission intensity and pH for SiCDs@SiO_2_-1 and SiCDs@SiO_2_-2.

As shown in Figure 15, the fluorescence intensity of the fluorescent microsphere suspensions was measured at different pH. Figure 15a,b shows that the fluorescence intensity of the microspheres decreased by 15–34% under different pH conditions, but the emission wavelength remained unchanged. No fluorescence burst was observed, and fluorescence intensity rose as the pH of the suspensions increased. Notably, high fluorescence intensity was maintained even in strongly acidic and alkaline solutions. The above results show that the fluorescent microspheres can exist stably in environments at different pH, which makes them promising for use as fluorescent markers under different conditions.

#### 2.3.3. Effect of Storage Time on the Emission Behavior of Microsphere Suspensions

**Figure 16:**
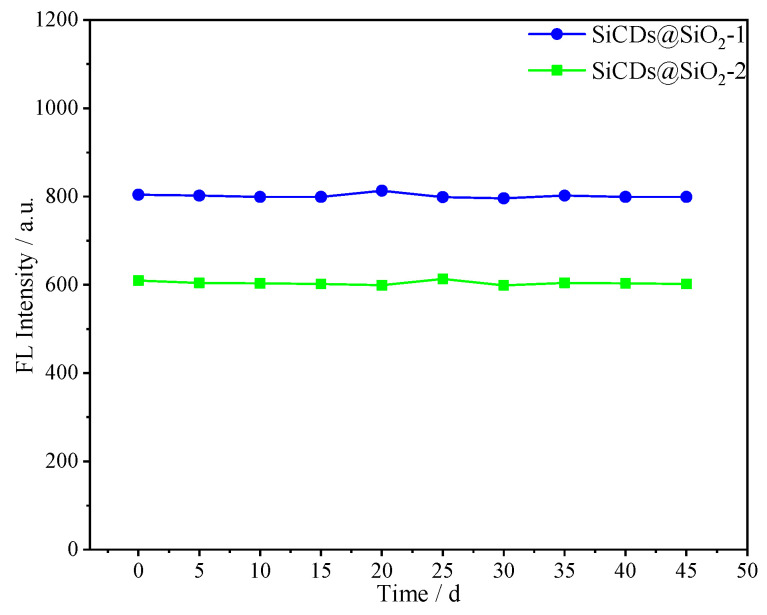
Fluorescence spectra of microspheres (0.2 mg/mL) in fracturing fluid aged for different periods (0–45 days).

Figure 16 displays the relationship between the fluorescence intensity of the microspheres and residence time in fracturing fluid. The fluorescence intensity of the fluorescent microsphere suspension remained almost unchanged during 45 days at 25 °C. These results show that the fluorescent microspheres remained stable in fracturing fluid for a long period of time, which implies that these stable microspheres might be potentially valuable as fluorescent markers in the complex environment of horizontal wells.

#### 2.3.4. Effect of Metal Ions on the Fluorescence Behavior of Microsphere Suspensions

**Figure 17:**
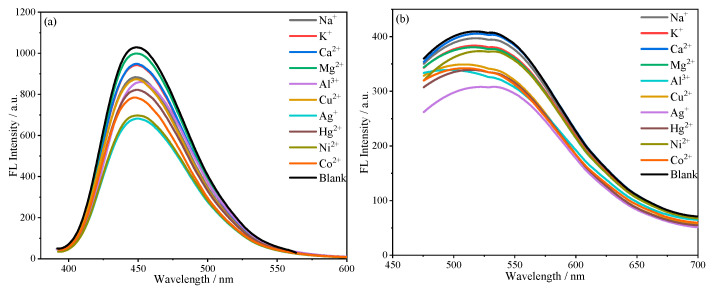
(**a**) The fluorescence intensity of SiCDs@SiO_2_-1 (0.2 mg/mL) in aqueous solutions containing different metal ions (10 mM). (**b**) The fluorescence intensity of SiCDs@SiO_2_-2 (0.2 mg/mL) in aqueous solutions containing different metal ions (10 mM).

We measured the fluorescence spectra of the microspheres in solutions of different metal ions. As shown in Figure 17a,b, the fluorescence intensity of the microspheres decreased when they were dispersed in solutions containing different metal ions (Na^+^, K^+,^ Ca^2+^, Mg^2+^, Al^3+^, Cu^2+^, Ag^+^, Hg^2+^, Ni^2+^, or Co^2+^). The effects of Ag^+^ and Ni^2+^ on SiCDs@SiO_2_-1 were the largest, decreasing the fluorescence intensity of SiCDs@SiO_2_-1 by about 40%, whereas the effects of other metal ions on the fluorescence intensity of SiCDs@SiO_2_-1 were small (Figure 17a). The effect of Ag^+^ on the fluorescence intensity of SiCDs@SiO_2_-2 was the largest, whereas the effects of other metal ions on the fluorescence intensity of SiCDs@SiO_2_-2 were minor (Figure 17b). However, regardless of the presence of interfering ions, SiCDs@SiO_2_-1 and SiCDs@SiO_2_-2 still displayed strong fluorescence that was detected by the spectrometer. Overall, the fluorescence signals of the microspheres were readily detected in the presence of various metal ions.

## 3. Experimental

### 3.1. Reagents and Instruments

CA, AEAPMS, triiron tetraoxide (Fe_3_O_4_) nanoparticles (200 nm), 1,3,5-homobenzenetricarboxylic acid, *p*-aminobenzenesulfonic acid, *o*-aminophenol, and tetraethyl orthosilicate (TEOS) were purchased from Shanghai Macklin Biochemical Technology Co., Ltd.(Shanghai, China); glucose and absolute ethanol (EtOH) were purchased from Tianjin Zhiyuan Reagent Co., Ltd.(Tianjin, China); *o*-phenylenediamine, anhydrous aluminum chloride (AlCl_3_), anhydrous cobaltous chloride (CoCl_2_), and nickel chloride (NiCl_2_) were purchased from Shanghai Aladdin Biochemical Technology Co., Ltd.(Shanghai, China); 8-aminoquinoline and 1,4-naphthoquinone were purchased from Bide Pharmatech Co., Ltd.(Shanghai, China); Thiourea and potassium chloride (KCl) were purchased from Beijing InnoChem Science & Technology Co., Ltd.(Beijing, China); Sodium hydroxide (NaOH), ammonium hydroxide (NH_3_∙H_2_O), anhydrous magnesium sulfate (Mg_2_SO_4_), and calcium chloride (CaCl_2_) were purchased from Sinopharm Chemical Reagent Co., Ltd.(); Sodium chloride (NaCl) was purchased from Tianjin Tianli Chemical Co., Ltd.(Tianjin, China); Copper(II) sulfate (Cu_2_SO_4_) was purchased from Tianjin Yongda Chemical Reagent Development Center(Tianjin, China); silver nitrate (AgNO_3_) was purchased from Chengdu Chemical Reagent Factory(Chengdu, China); mercury(II) chloride (HgCl_2_) was purchased from Jiangyan Global Chemical Factory(Taizhou, China). Deionized water was prepared in our laboratory.

An analytical balance (Beijing Sartorius Scientific Instruments Co., Ltd., Beijing, China), rotary evaporator (Shanghai Yuezhong Instrument Co., Ltd., Shanghai, China), low-temperature cooling water circulating pump (Gongyi Yuhua Instrument Co., Gongyi, China), intelligent magnetic stirrer (Gongyi Yuhua Instrument Co., Gongyi, China), circulating water vacuum pump (Gongyi Yuhua Instrument Co., Gongyi, China), vacuum drying oven (Tianjin Gongxing Laboratory Instrument Co., Tianjin, China), and ultraviolet (UV) analyzer (Shanghai Heqi Chemical Technology Co., Shanghai, China) were used in the preparation of SiCDs@SiO_2_.

The SiCDs@SiO_2_ microspheres were characterized using a fluorescence spectrophotometer (LS55, PerkinElmer, Waltham, MA, USA), Fourier-transform infrared (FTIR) spectrometer (Nicolet6700, ThermoNicolet Instrument Co., Madison, WI, USA), UV–visible (vis) spectrophotometer (PE Lambda650, PerkinElmer, Waltham, MA, USA), powder X-ray diffractometer (Empyream, Spectris Instrumentation and Systems Shanghai Ltd., Shanghai, China), X-ray photoelectron spectrometer (ThermoFisher Nexsa, Thermo Fisher Scientific Inc., Waltham, MA, USA), field-emission scanning electron microscope (FESEM; MIRA3, TESCAN CHINA, Shanghai, China), high-resolution laser confocal microscope (TCS SP8, Leica Microsystems, Wetzlar, Germany), low-speed equilibrium centrifuge (LDZ4-1.8, Beijing Leibel Medical Instrument Co., Ltd., Beijing, China), and a magnetic hyperthermia reaction device (GP-20, Suzhou Hongchuang High Frequency Heating Equipment Co., Ltd., Suzhou, China).

### 3.2. Synthesis Method

#### 3.2.1. Synthesis of Water-Soluble SiCDs

SiCDs-1 was prepared by magnetic hyperthermia using CA and AEAPMS as raw materials. CA (1 g) and Fe_3_O_4_ nanoparticles (200 nm, 30 wt%) were thoroughly ground using a mortar and pestle. The ground powder was transferred to a 100 mL pear-shaped flask. AEAPMS (2 mL) was added, and then the mixture was stirred until it became uniform. The flask was placed on a magnetic hyperthermia reaction device and reacted for 1.5 min. After the reaction, the prepared SiCDs-1 was dissolved in deionized water, centrifuged to remove the Fe_3_O_4_ nanoparticles, and then the solvent was removed using a rotary evaporator to obtain a yellow-brown oily liquid, which was dried in a vacuum drying oven. The dried CDs were a light yellow-brown gel-like solid.

SiCDs-2 was prepared by magnetic hyperthermia using CA, glucose, and AEAPMS. CA (1 g) and glucose (1 g) were ground with Fe_3_O_4_ nanoparticles (200 nm, 30 wt%) using a mortar and pestle. The ground powder was transferred to a 100 mL pear-shaped flask and AEAPMS (4 mL) was added. The mixture was stirred well and then placed on the magnetic hyperthermia reaction device for 1.5 min. After the reaction, the prepared SiCDs-2 was dissolved in deionized water and centrifuged to remove the Fe_3_O_4_ nanoparticles. The solvent was removed by rotary evaporation to obtain a yellow-brown oily liquid, which was dried in a vacuum drying oven to give the dried CDs as a brown gel-like solid.

Using the procedure described above, SiCDs-3 was prepared using CA, 1,3,5-homobenzenetricarboxylic acid, and AEAPMS; SiCDs-4 was prepared using CA, *o*-phenylenediamine, and AEAPMS; SiCDs-5 was prepared using CA, *o*-aminophenol, and AEAPMS; SiCDs-6 was prepared using CA, *p*-aminobenzenesulfonic acid, and AEAPMS; SiCDs-7 was prepared using CA, 8-aminoquinoline, and AEAPMS; SiCDs-8 was prepared using CA, thiourea, and AEAPMS; SiCDs-9 was prepared using CA, 1,4-naphthoquinone, and AEAPMS.

#### 3.2.2. Synthesis of Water-Soluble SiCDs@SiO_2_ Spheres and Pure SiO_2_ Spheres

Anhydrous EtOH (60 mL), NH_3_∙H_2_O (10 mL), deionized water (3 mL), and TEOS (8 mL) were added to a 250 mL three-necked flask and then heated at 55 °C for 10 min. SiCDs-1 (0.2 g) dispersed in deionized water (3 mL) was added to the reaction system, which was then heated at 55 °C for a further 3 h. The resulting fluorescent microspheres (SiCDs@SiO_2_-1) were isolated by centrifugation, washed three times with EtOH, and then dried in a vacuum drying oven.

Using the procedure described above, SiCDs-2, SiCDs-3, SiCDs-4, SiCDs-5, SiCDs-6, SiCDs-7, SiCDs-8, and SiCDs-9 were included in fluorescent microspheres, which are denoted as SiCDs@SiO_2_-2, SiCDs@SiO_2_-3, SiCDs@SiO_2_-4, SiCDs@SiO_2_-5, SiCDs@SiO_2_-6, SiCDs@SiO_2_-7, SiCDs@SiO_2_-8, and SiCDs@SiO_2_-9, respectively.

As a control sample, SiO_2_ microspheres were prepared without adding SiCDs during the above reaction.

#### 3.2.3. Preparation of RSiCDs

SiCDs@SiO_2_-1 and SiCDs@SiO_2_-2 were dispersed in 2 M NaOH to form suspensions with a concentration of 0.2 mg/mL. The suspensions were aged overnight at room temperature. After the samples dissolved, the suspensions changed from turbid to clear, providing RSiCDs-1 and RSiCDs-2 solutions.

### 3.3. Calculation of QY

The QY of SiCDs was measured using quinine sulphate dihydrate, which has a QY of 54% in 0.1 M sulfuric acid (H_2_SO_4_), as the standard reference material. The CDs and quinine sulphate dihydrate were diluted to several different concentrations with deionized water (refractive index 1.33) and 0.1 M H_2_SO_4_ (refractive index 1.33), respectively. The absorbance at 360 nm and integrated fluorescence intensity at an excitation wavelength of 360 nm were measured for each solution. To suppress the self-absorption effect, the UV–vis absorption intensities of SiCDs and quinine sulphate dihydrate solutions were kept below 0.1.

The QY of SiCDs was calculated according to the following equation:

$${\mathrm{QY}}_{x}={\mathrm{QY}}_{s}\frac{K_{x}}{K_{s}}\times {\left(\frac{\eta_{x}}{\eta_{s}}\right)}^{2}$$

where η is the refractive index of the solvent, K is the slope of the linear fitting of the integrated fluorescence intensity to the absorbance, and the subscripts s and x represent the reference standard (quinine sulphate) and CD sample, respectively [29].

### 3.4. Performance Testing of Water-Soluble SiCDs@SiO_2_ Spheres

The water-soluble SiCDs@SiO_2_ synthesized and prepared in this study theoretically possess the properties of excitation wavelength independence and environmental stability. It is presumed that it can be applied as a fluorescent tracer to locate the water breakthrough section in horizontal wells. Currently, the issue of water breakthrough in horizontal wells has become a common technical bottleneck in the development of low-permeability reservoirs. Existing water detection technologies struggle to quickly and accurately identify the location of water breakthrough in horizontal wells [30]. To explore novel solutions, this study conducted performance tests on SiCDs@SiO_2_ to verify its potential applicability as a fluorescent tracer in horizontal well water detection, thus providing valuable reference and basis for subsequent research in related fields.

#### 3.4.1. Performance Testing of Thermal Stability of Microsphere Suspensions

When microspheres are used as fluorescent markers for water detection in horizontal wells, they usually flow with the oil supply, which requires them to possess high stability. To evaluate their potential for use as fluorescent markers for oil-field applications, the thermal stability of the fluorescent microspheres was investigated. The fluorescent microspheres were added to fracturing fluid at a dosage of 0.2 mg/mL and then heated at different temperatures (25, 60, 90, 120, and 150 °C) for 1 h. After cooling to room temperature, fluorescence emission spectra were recorded.

#### 3.4.2. Performance Testing of Effect of pH on Microsphere Suspensions

Solution pH is an important factor affecting the stability of fluorescent dyes. The complex formation conditions during oil and gas field development require fluorescent markers to adapt to environments with different pH. Therefore, the fluorescence intensity of microsphere suspensions at different pH was measured. The pH of aqueous microsphere suspensions (0.2 mg/mL) was adjusted from 1–14 using NaOH or hydrochloric acid, and then fluorescence emission spectra were collected.

#### 3.4.3. Performance Testing of Effect of Storage Time on the Stability of Microsphere Suspensions

When fluorescent markers are injected into the strata along with fluid, it usually takes some time before the reservoir information is brought back to the surface. If the markers stay in the complex drilling fluid system for a long time, they may precipitate, which will directly affect their fluorescence intensity. For this reason, the relationship between fluorescence intensity and the residence time of fluorescent microspheres in fracturing fluid was investigated. A suspension of fluorescent microspheres was left under natural conditions for 45 days. Fluorescence emission spectra of the microsphere suspension were recorded every 5 days.

#### 3.4.4. Performance Testing of Effect of Metal Ions on the Stability of Microsphere Suspensions

Strata usually contain some metal ions, which may affect the fluorescence intensity of potential markers. For this reason, the fluorescence intensity of microsphere suspensions in the presence of different ions (Na^+^, K^+^, Ca^2+^, Mg^2+^, Al^3+^, Cu^2+^, Ag^+^, Hg^2+^, Ni^2+^, and Co^2+^) was evaluated. Solutions of interfering substances (10 mM) were prepared with deionized water, and then fluorescent microspheres (0.2 mg/mL) were added to obtain test solutions. The excitation wavelength for fluorescence intensity measurements was set to 360 nm.

## 4. Conclusions

In summary, a series of SiCDs were prepared simply and efficiently by the magnetic hyperthermia method. Compared with conventional reactions to form CDs, the magnetic hyperthermia reaction has high energy, a fast temperature rise, favorable temperature stability, uniform heating, high safety, and high efficiency. Our simple approach can achieve gram-scale preparation of SiCDs in a short time. The obtained SiCDs displayed promising fluorescence characteristics, including high water solubility, the capacity to emit light in both solution and the solid state, and excitation wavelength-independent properties. The obtained SiCDs were readily embedded in SiO_2_ spheres by inclusion in the Si-O network during the Stöber reaction, providing fluorescent microspheres loaded with SiCDs.

Nine kinds of fluorescent microspheres were prepared, of which SiCDs@SiO_2_-1 was spherical with a smooth surface and uniform size of about 500 nm and displayed blue fluorescence. Meanwhile, SiCDs@SiO_2_-2 particles were spherical with a smooth surface and uniform size of about 400 nm and exhibited green fluorescence, indicating that the SiCDs were embedded in the SiO_2_ spheres. Each microsphere acted as an independent luminescent body, making them suitable for use as fluorescent markers. The SiCDs@SiO_2_ microspheres can be considered to possess a sandwich structure consisting of an inner SiO_2_ sphere, a middle SiO_2_/SiCDs layer, and an outermost SiO_2_ shell layer. The SiCDs@SiO_2_ microspheres have size controllability and favorable water solubility with tunable structure and fluorescence and excitation wavelength-independent properties.

The SiCDs@SiO_2_ microspheres were stable up to a temperature of 150 °C, and their fluorescence was detected at different pH and in the presence of different metal ions and remained stable in fracturing fluid during long-term monitoring for 45 days. Therefore, the water-soluble SiCDs@SiO_2_ microspheres have favorable temperature, acid, and alkali resistance. Furthermore, the storage time and different metal ions had minimal influence on the fluorescence signal of the microsphere suspensions. The results of these performance tests indicate that the prepared SiCDs@SiO_2_ are detectable, water soluble, and stable. These properties indicate that SiCDs@SiO_2_ can be applied as a fluorescent labeling probe in complex horizontal well environments, with potential applications in the field of horizontal well water detection.

## Data Availability

The data that support the findings of this study are available from the corresponding author upon reasonable request.
